# Some Questions to Sir Napier Burnett

**Published:** 1920-10-16

**Authors:** 


### POOR-LAW HOSPITALS UNDER VOLUNTARY COMMITTEES.

### Some Questions to Sir Napier Burnett.

We have already alluded to the suggestion for placing the Poor-law infirmaries under voluntary committees as a means whereby the State resources and the voluntary spirit could complement each other; but the suggestion as first put forward hung somewhat in the air. Some further light is thrown upon the practical side of the proposal in an interview which Sir Napier Burnett has given to a representative of the *Newcastle Chronicle*. Sir Napier began by saying that while the present provision made by the voluntary hospitals for patients and staff is in many cases inadequate, the hospitals have no money to pay for further extensions. It is to provide the needed accommodation, he said, that the Ministry of Health (Miscellaneous Provisions) Bill is designed. Clause 2 will empower county and borough councils to supply and maintain hospitals; to contribute to voluntary hospitals within their area, and to undertake the maintenance of any Poor-law hospitals and infirmaries within their boundaries. Since it has been urged that many partially occupied Poor-law infirmaries should be converted into general hospitals to relieve the latter of congestion and their waiting cases, how, asked Sir Napier Burnett, is it proposed to maintain these adapted institutions? He replied that Dr. Addison must obtain the necessary financial powers, and that the only local Government machinery which he can use is the county and borough councils, because, whatever the technical powers conferred by the Public Health Act of 1875, these, in practice, are restricted to hospitals for infectious diseases. The provision and maintenance of general hospitals has been left by public preference to voluntary enterprise.

### The Present Proposal.

If the present proposal were carried out, he continued, you would have the voluntary and municipal hospitals side by side with general hospitals, treating similar cases, and can it be doubted that subscriptions to the voluntary hospitals will then disappear when the philanthropic public realises that by continuing to subscribe they are relieving the rates? If this were a fair deduction, he thought that legacies also will be cancelled, and that the two types of hospitals conducted on the same lines, but by different financial means, cannot continue to exist.

A general hospital on the rates could be, he felt sure, successful and efficient. Why, then, not have municipally controlled general hospitals? General hospitals can be maintained on a voluntary basis, or by the State, or by the municipality. The voluntary principle is not an end in itself, but a means, and even more important than the voluntary principle is the necessity for adequate treatment of the sick persons. It is for the public to decide which they prefer. To make a right choice they should know all the facts. The first is that municipal hospitals, to be run as efficiently as the voluntary hospitals, will cost at least one-third more, at a moderate estimate. Voluntary hospital committees can buy necessary commodities cheaper than State or municipal bodies. The inquiry conducted into hospital buying by the North-East Hospitals Association last year proved this. It also supported the recent assertion of a medical officer of health that "the corporation cannot purchase what is required so cheaply as private agencies." The efficiency of the municipal or State hospital is not in question. The question is with an unprecedented burden of debt, and with local rates at their present unexampled height, whether a general policy of putting hospitals on the rates can be afforded. The Government will have to face this question.

### The Possible Solution.

The solution, in his view, is that use should be made of the Poor-law hospitals, whose great need is more beds. Voluntary finance cannot tackle the provision of these, but the Ministry of Health is in possession of Poor-law hospital buildings, which in many cases are adequate. He therefore urged that these buildings should be handed over to voluntary committees to be managed as voluntary hospitals. This plan seems to him to bridge the difficulty. Since Dr. Addison, in the House of Commons, has repeatedly expressed his desire to preserve the voluntary hospitals, Sir Napier offered the above suggestion as a practical policy and the form which a State subsidy should take. The Government by this means will provide the necessary buildings, and the voluntary committees their economical and personal management. The Government, he thought, may also reasonably be asked to pay for the training of nurses and for the medical registrars, whose duties largely consist of keeping the records, which are of great service to the Ministry.

That is Sir Napier Burnett's outline of his scheme. As sketched, it has its attractive side, but the points most interesting to the voluntary system are still far from clear. In the first place, where are these voluntary committees to come from? are the Poor-law hospitals to be affiliated to the great voluntary hospitals in their neighbourhood, and managed as annexes from there? If not, where are the committees to be found with the necessary training and experience? Presumably the former alternative is intended. If so, the responsibilities of the hospital boards of management would be vastly increased, and complicated by the fact that they would be administering State funds as well as their own. Again, to State-supported institutions patients would presumably have free access, except in special cases, and this might affect the position of voluntary hospitals which are imposing a charge towards the cost of patients' maintenance.

Further, supposing the popularity of the volun-

**P.-L. Hospitals under Voluntary Committees**—(*cont.*).

tarily supported hospital were found to be set against the free treatment and maintenance provided in the State hospital under voluntary management, then the effect might be that one institution would secure one class and another another, so that the poor would mainly flock to the State institution and the paying patient to the voluntary. Again, once the voluntary hospitals began to administer institutions supported out of State or municipal funds, the public might still feel Sir Napier's own objection that by subscribing they were indirectly relieving the rates. For the funds of one half of the work would be public funds, whoever administered them; and this might also have the effect of undermining the economy of voluntary management. Again, would the voluntary spirit survive in the shackles of State supplies, when every expense would have to be officially checked and scrutinised? The suggestion is that a voluntary committee would harmonise State finance. Might not the influence be reversed, and the bureaucratic spirit invade the voluntary committee? We ask these questions to gain more light on a plan so faintly, if alluringly, sketched on paper. Finally, is the question to be considered wholly in the light of finance? In other words if, as we know, the voluntary spirit is in itself, and more than Sir Napier seems to admit, if not an end, at least a virtue, must one not be prepared to pay for that virtue in the difficulties peculiar to its exercise? The chief is that it cannot provide sufficient money to cover the whole ground, though that which it does cover is magnificently cultivated. The State is in the opposite position. Its resources are almost unlimited, but its spirit is weak. The solution appears to be not to provide the voluntary system with institutions entirely supported by State funds, but to provide the institutions, in lieu of a grant of capital, and to pay the voluntary committees entrusted with them for the work done, and the particular class of cases treated there. If there is not some infusion of voluntary finance, however limited, in the municipal hospitals to be entrusted, as Sir Napier's plan, to the management of voluntary committees, it is doubtful if the voluntary spirit will survive contact with officialdom.

